# Supplementary material for: Domain 2 of Hepatitis C Virus Protein NS5A Activates Glucokinase and Induces Lipogenesis in Hepatocytes
Source: Int J Mol Sci. 2022 Jan 14;23(2):919. doi: 10.3390/ijms23020919 (PMC8780509; doi:10.3390/ijms23020919)

Figure S1: Analysis of NS5A-D2 expression in transduced Huh7-*GCK*$^+$/*HK2*$^-$ cells

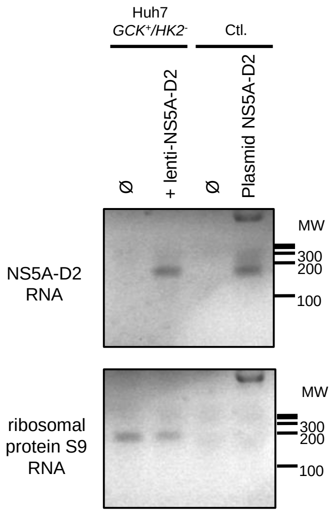

Figure S2: ECAR and OCR quantification during Glycolytic Rate Test performed at 5 and 25mM glucose, on Huh7-*GCK*$^{+}$/*HK2*$^{-}$ cells expressing or not NS5A-D2.

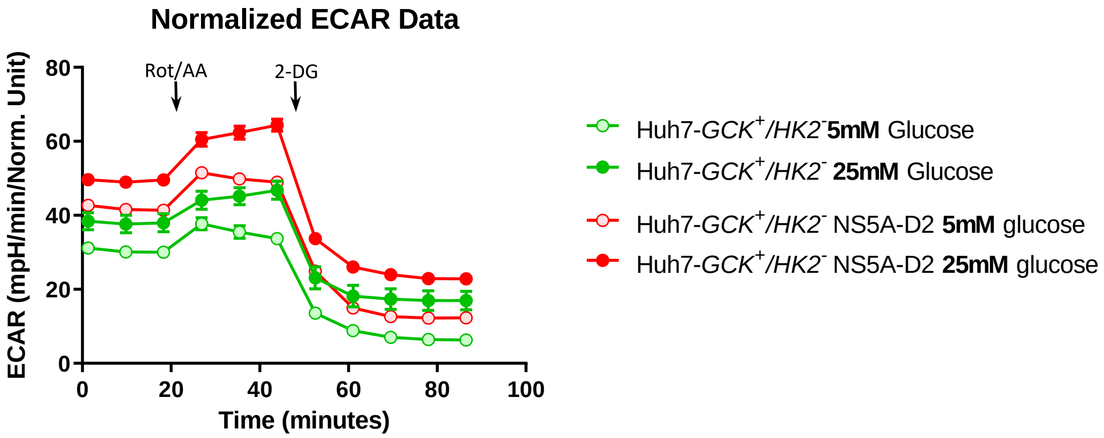


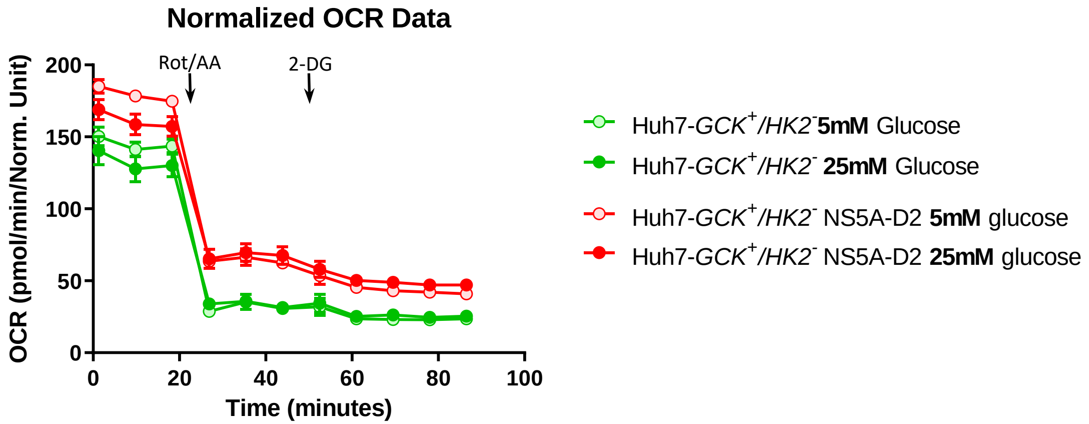

Figure S3: NS5A-D2 alters allosteric properties of GCK enzyme

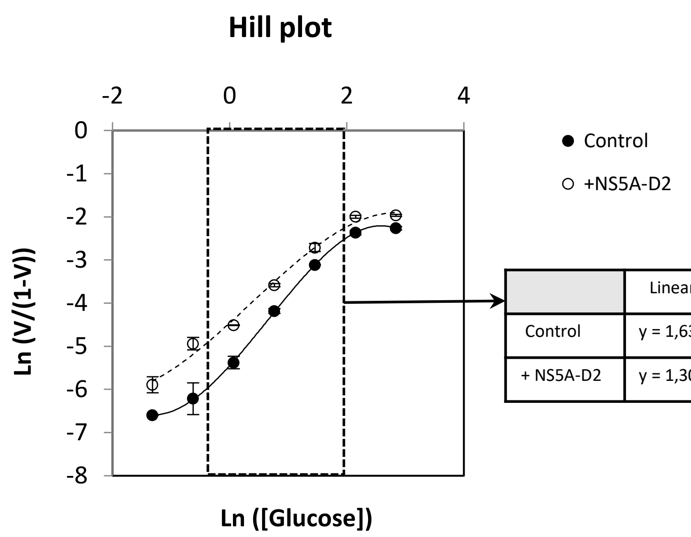


| | Linear regression | r² | Hill coef. |
|---|---|---|---|
| Control | y = 1,6335x - 5,4735 | 0,9989 | 1.63 |
| + NS5A-D2 | y = 1,3003x - 4,5971 | 0,9997 | 1.30 |

Figure S4: Western-blot Figure 1A

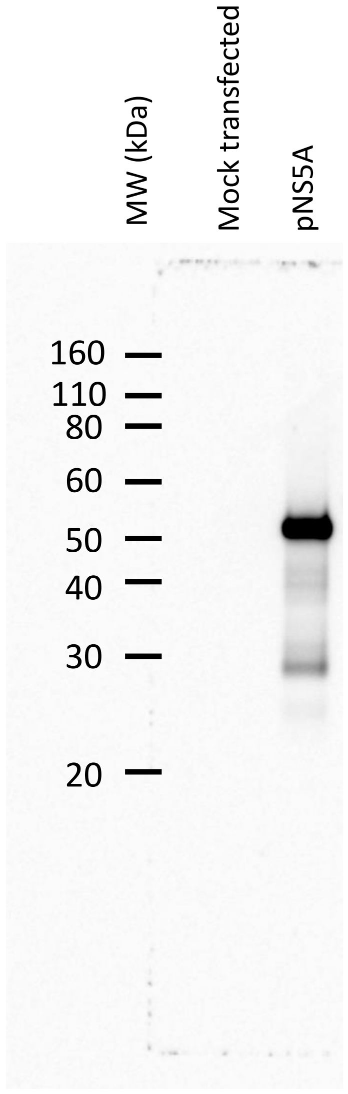

# Figure S5: Western-blots Figure 4A

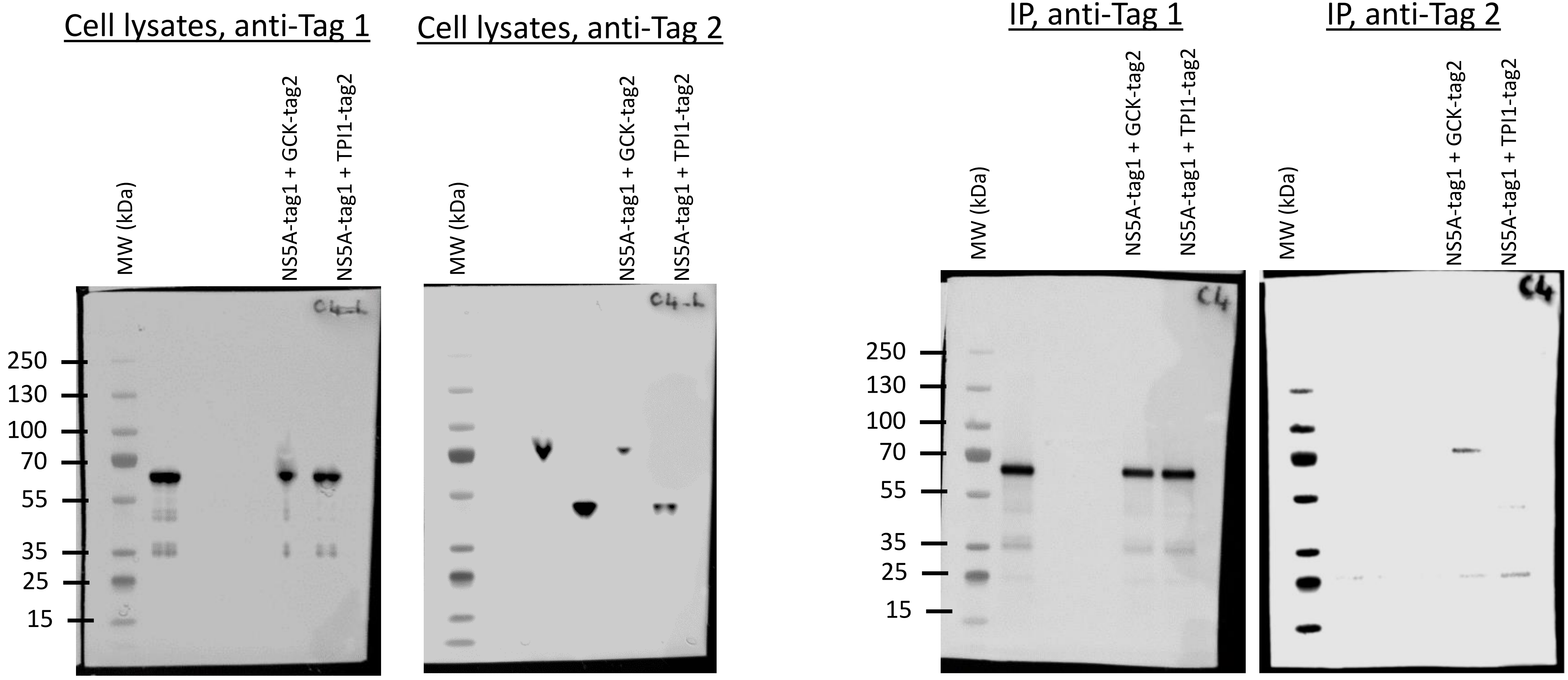

Supplement: Supplementary file 1 [file ijms-23-00919-s001.zip › ijms-1526520-supplementary.pdf]
